# An Evolutionary-Algorithm-Driven Efficient Temporal Convolutional Network for Radar Image Extrapolation

**DOI:** 10.3390/biomimetics11020122

**Published:** 2026-02-06

**Authors:** Peiyang Wei, Changyuan Fan, Yuyan Wang, Tianlong Li, Jianhong Gan, Can Hu, Zhibin Li

**Affiliations:** 1 School of Software Engineering, Chengdu University of Information Technology, Chengdu 610225, China; weipy@cuit.edu.cn (P.W.); yuyanwang755@gmail.com (Y.W.); tianlongl278@gmail.com (T.L.); gjh@cuit.edu.cn (J.G.); hucan028@outlook.com (C.H.); 2 Chongqing Institute of Green and Intelligent Technology, Chinese Academy of Sciences, Chongqing 400714, China; 3 Automatic Software Generation & Intelligence Service Key Laboratory of Sichuan Province, Chengdu 610225, China; 4 College of Electronic Engineering, Chengdu University of Information Technology, Chengdu 610225, China

**Keywords:** radar extrapolation, adaptive hyperparameter optimization, associated algorithm, convolutional neural network, evolutionary algorithm, deep learning

## Abstract

Radar image extrapolation serves as a fundamental methodology in meteorological forecasting, facilitating precise short-term weather prediction through spatiotemporal sequence analysis. Conventional approaches remain constrained by progressive image degradation and artifacts, substantially limiting operational forecasting reliability. This research introduces E-HEOA—an enhanced deep learning architecture with integrated hyperparameter optimization. Our framework incorporates two fundamental innovations: (a) a hybrid metaheuristic optimizer merging a Gaussian-mutated ESOA and Cauchy-mutated HEOA for autonomous learning rate and dropout optimization and (b) embedded ConvLSTM2D modules for enhanced spatiotemporal feature preservation. Experimental validation on 170,000 radar echo samples demonstrates superior performance, demonstrating considerable enhancement in almost all aspects relative to the baseline model while establishing new state-of-the-art benchmarks in prediction fidelity, convergence efficiency, and structural similarity metrics.

## 1. Introduction

Radar imagery is one of the important materials for meteorological observation, which can capture real-time changes in atmospheric elements like precipitation and clouds. By analyzing and processing radar images [1,2,3,4,5], it is possible to predict future weather conditions. However, due to extensive information in high-resolution radar images, it is difficult for conventional image processing methods to effectively extract useful data, thus restricting the accuracy and clarity of radar image extrapolation. How to fully utilize the information in radar images to enhance extrapolation accuracy and clarity has become one of the hot issues in the field of weather forecasting [6,7,8,9,10,11,12,13,14,15].

In the field of radar image extrapolation, numerous advanced convolutional methods have been proposed, primarily focusing on architectural innovations of deep neural networks to capture spatiotemporal features. Shi et al. proposed a ConvLSTM2D-CNN method [8], which consists of a recurrent dynamic subnetwork and a probabilistic prediction layer. This method constructs a recurrent structure into the convolutional layers. However, it may produce a blurring effect during prediction by learning multiple patterns, which leads to less accurate results. Wang et al. presented a two-stage extrapolation method based on a 3D convolutional neural network and conditional generative adversarial network [9], which can correct echo intensity and enhance echo details. However, it still faces challenges when handling high-dimensional spatiotemporal data, especially in accurately predicting radar echolocation and intensity during adversarial training. Yang et al. developed a radar extrapolation method based on a self-attention mechanism and long short-term memory (LSTM) network [10], which preserves global spatiotemporal features in the original Spatiotemporal LSTM (ST-LSTM) and obtains a Self-Attention Integrated ST-LSTM Recurrent Unit (SAST-LSTM), thus capturing two global spatiotemporal features of radar echo movement. In addition, the self-attention mechanism effectively captures global features; however, it suffers from high computational complexity, especially for high-resolution radar echo data, which may reduce time efficiency by processing large datasets, thus restricting its real-time applications. Zheng et al. designed a GAN-argcPredNet v2.0 method that suppresses attenuation by avoiding blurring and maintaining intensity [11]. The Spatiotemporal Image Correlation (STIC) prediction network is designed as the model generator. By suppressing the blurring effect of rainfall distribution and reducing the negative bias in STIC attention, the generator produces accurate images. However, this method is a variant of GAN: the adversarial training between the generator and discriminator requires the Nash equilibrium, which is difficult to perfectly realize in practice. Therefore, this method may face instability and fluctuations during training. Additionally, Liu et al. explored robust feature representation learning in complex scenes [12], and Wang et al. investigated the preservation of structural consistency in multidimensional data under geometric transformations [13]. These two works share methodological commonalities with the stable spatiotemporal modeling and complex evolution pattern prediction of radar echo sequences studied in this paper, thus providing valuable references for the design and robustness analysis of the radar extrapolation model.

Most of the aforementioned methods primarily focus on the design and optimization of deep learning models. While improvements in model architecture can indeed enhance prediction accuracy to some extent, the extent of such improvement is limited. Particularly for models that incorporate attention mechanisms, which have garnered widespread attention in recent years, there is a qualitative advancement in structure compared to traditional models. However, this progress inevitably leads to an increase in computational complexity, which proves to be more detrimental than beneficial for real-time prediction applications that demand extremely high timelines. On the other hand, research that relies solely on traditional models with structural modifications for radar image prediction, while avoiding an increase in computational complexity, suffers from a narrow focus on model architecture alone. The potential enhancement in prediction accuracy is severely constrained by the limited scope of the research. In contrast, improving model hyperparameters presents a promising and underexplored direction for existing real-time radar image prediction studies. However, hyperparameters need to be set in advance in deep learning models, such as learning rate and regularization strength. They have a significant influence on the training process and the final performance of the model. By optimizing hyperparameters, this study aims to find the most suitable model configuration for a specific dataset and problem, thereby significantly improving model performance [14]. Therefore, it is evident that the performance of deep learning models is closely related to hyperparameter configuration. However, hyperparameter adjustment is frequently done manually. Manual hyperparameter tuning involves selecting appropriate combinations based on experience and intuition through multiple experiments and adjustments to optimize model performance. However, this process is time-consuming and labor-intensive, and it also suffers from local optima and overfitting. Hence, automated and efficient hyperparameter optimization methods are particularly important. Optimizing the hyperparameters of the model for radar image extrapolation tasks is the core to enhancing model performance and image clarity [16,17,18,19,20,21,22,23,24,25].

Current primary hyperparameter tuning methods, aside from the manual tuning approach relying on the experimenter’s experience mentioned above, also include traditional tuning algorithms represented by random search, grid search, and Bayesian optimization, as well as evolutionary algorithm tuning. Among traditional tuning algorithms, random search is significantly limited in optimization efficiency and effectiveness due to its enumeration-based tuning approach. While grid search improves upon random search, it still suffers from severely low tuning efficiency. Bayesian optimization, although outperforming random search and grid search in some optimization tasks, is limited in high-dimensional spaces, parallel computing, and scenarios with extremely low evaluation costs. Additionally, it is sensitive to initial points and may converge to local optima. In contrast to the above tuning algorithms, the advantage of evolutionary algorithms in hyperparameter tuning lies in their population-based parallel exploration, independence from gradient information, ability to effectively handle high-dimensional, discontinuous, black-box problems, and utilization of crossover and mutation to escape local optima and explore solution spaces with greater potential.

As for a technique for automatically tuning hyperparameters, evolutionary algorithms have significant advantages in model hyperparameter optimization [15,26,27]. So far, several new evolutionary algorithms have been applied in the field of deep learning, such as ant colony optimization [16], gray wolf optimization [17] and whale optimization [18]. The ant colony optimization algorithm frequently gets stuck in the local optimum during the search process. The gray wolf optimization algorithm simulates the leadership hierarchy and hunting mechanism of gray wolves in nature; however, it also obtains local optimum [9,28,29,30,31,32,33,34,35,36]. The whale optimization algorithm also faces issues of unstable solution and local optimum. In summary, these above models with a single optimization algorithm have two drawbacks:

(a) A single optimization algorithm tends to trap the entire neural network in a local optimum during the search process, thus limiting the improvement of image clarity;
(b) The search mechanism of a single optimization algorithm may lead to slower convergence in later stages during the AS iteration process, which would limit the searching efficiency of the optimal solution.

For addressing these hyperparameter optimization issues, instead of proposing yet another standalone evolutionary algorithm, this paper introduces a mutation-enhanced associated optimization framework. The associated optimization algorithm has shown vital virtues [19,20]. It can handle complex constraints and diverse hyperparameter spaces by simultaneously optimizing multiple hyperparameters. This method reduces the time cost of tuning each hyperparameter individually. By comprehensively considering the interactions between multiple hyperparameters and their effect on model performance, these models enhance the generalization ability to search for the globally optimal hyperparameter combination. It allows for the efficient exploration of the hyperparameter space and improves optimization efficiency [37,38,39,40,41,42,43,44,45,46].

More importantly, this paper innovatively proposes the E-HEOA method, which combines two evolutionary algorithms with a unified framework. It aims to integrate the egret swarm optimization algorithm (ESOA) [22] based on Gaussian mutation with the human evolutionary optimization algorithm (HEOA) [26] based on the Cauchy mutation. The main innovations of this paper are as follows:

(a) The Gaussian mutation-based ESOA and Cauchy mutation-based HEOA are applied to optimize the hyperparameters of ConvLSTM2D. The enhanced algorithms with mutation operations further strengthen the global search capability, thereby effectively improving the superiority of radar prediction images.
(b) The adaptive optimization approach replaces manual tuning based on experience. On this foundation, a joint framework is proposed to replace single optimization algorithms, positively influencing image fidelity.
(c) Extensive experiments are conducted on a radar extrapolation dataset with 170,000 data points. The experimental results indicate that the proposed E-HEOA method achieves high prediction performance, convergence speed and fitting accuracy, thus effectively handling radar extrapolation issues.

The rest of this paper is as follows: Section 2 presents the related evolutionary optimization algorithms, Section 3 discusses the E-HEOA, and Section 4 gives experiment and results analysis. Lastly, conclusions are given in Section 5.

## 2. Preliminaries

### 2.1. Egret Swarm Optimization Algorithm

Inspired by the sit-and-wait strategy of the egret and the attack strategy of the great egret, Chen et al. proposed the egret swarm optimization algorithm (ESOA), which integrates the advantages of the two strategies; it also analyzes the behaviors by mathematical modeling [24]. The ESOA consists of three basic components: the sit-and-wait strategy, the attack strategy, and the discriminant condition. These three components are described as follows:

(1) Sit-And-Wait Strategy

The learning rule of the sit-and-wait strategy is written as follows:

(1) $$loc_{goal\_est\_i}=\alpha (loc_{self\_i}),$$

where $\alpha \left(*\right)$ is the egret’s estimation method for the potential presence of prey at the current location, $loc_{self\_i}$ represents the egret’s current position, and $loc_{goal\_est\_i}$ is the estimated value of the prey at the current location [24].

(2) $$loc_{goal\_est\_i}=w_{i}\cdot loc_{self\_i},$$

where $w_{i}$ is the weight of the estimation method. $w_{i}$ is a random number between 0 and 1, which controls the $loc_{goal\_est\_i}$ corresponding to each $loc_{self\_i}$.

Based on (2), we define the error as follows:

(3) $$e_{i}=\;||loc_{goal\_est\_i}-loc_{goal\_i}|{|}^{2}/2.$$

In addition, the gradient and its direction are calculated by

(4) $$\begin{array}{l} {\hat{g}}_{i}=\frac{\partial {\hat{e}}_{i}}{\partial w_{i}}\\ =\frac{\partial ||loc_{goal\_est\_i}-loc_{goal\_i}{\left|\right|}^{2}/2}{\partial w_{i}}\\ =(loc_{goal\_est\_i}-loc_{goal\_i})\cdot loc_{self\_i}, \end{array}$$

(5) $${\hat{d}}_{i}={\hat{g}}_{i}/|{\hat{g}}_{i}|.$$

**Figure 1:**
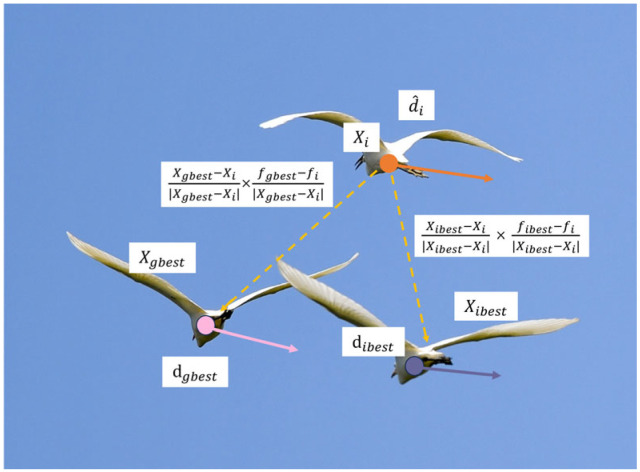
The direction correction mechanism of the optimization algorithm for an egret swarm.

During the egret’s predation process, it adjusts the position by referencing both the optimal egret position and its own position, which is shown in Figure 1. $d_{h,i}\in R_{n}$ represents the directional correction towards the best position of the squad; $d_{g,i}\in R_{n}$ is the directional correction towards the best position of all squads.

(6) $$d_{h,i}=\frac{loc_{selfbest\_i}-loc_{self\_i}}{\left|loc_{selfbest\_i}-loc_{self\_i}\right|}\cdot \frac{f_{best\_i}-f_{i}}{\left|loc_{selfbest\_i}-loc_{self\_i}\right|}+d_{best\_i},$$

(7) $$d_{g,i}=\frac{loc_{selfbest\_g}-loc_{self\_i}}{\left|loc_{selfbest\_g}-loc_{self\_i}\right|}\cdot \frac{f_{best\_g}-f_{i}}{\left|loc_{selfbest\_g}-loc_{self\_i}\right|}+d_{best\_g}.$$

(8) $$g_{i}=(1-t_{h}-t_{g})\cdot {\hat{d}}_{i}+t_{h}\cdot d_{h,i}+t_{g}\cdot d_{g,i},$$

where $g_{i}$ is gradient, $t_{h}\in [0,0.5],t_{g}\in [0,0.5]$.

In addition, adaptive weight is computed by

(9) $$v_{i\;}=\beta_{1}\;\cdot v_{i}+(1-\beta_{1}\;)\cdot g_{i},$$

(10) $$u_{i\;}=\beta_{1}\;\cdot u_{i}+(1-\beta_{1})\cdot g_{i}^{2},$$

(11) $$w_{i}=w_{i}\;-\;\frac{v_{i}}{\sqrt{u_{i}\;}}.$$

The next sampling location for egret A is given as

(12) $$loc_{anext\_i\;}=loc_{self\_i}+step_{a}\cdot \exp\left[\frac{-t}{\left(0.1\cdot iter_{\max}\right)}\right]\cdot hop\cdot g_{i},$$

(13) $$fit_{anext\_i}=f(loc_{anext\_i\;}),$$

where iter and $iter_{\max}$ represent the current iteration and the maximum iteration, respectively. hop denotes the gap between the lower and upper bounds of the solution space, and $fit_{anext\_i}$ is the fitness of $loc_{anext\_i\;}$.

(2) Attack Strategy

Egret B tends to randomly search for prey; its behavior is as follows:

(14) $$loc_{bnext\_i}=loc_{self\_i}\;+step_{b}\;\cdot tan(t_{b,i}\;)\cdot hop/(1+iter),$$

(15) $$fit_{bnext\_i}\;=f(loc_{bnext\_i}),$$

where $t_{b,i}$ is an iterative random number, and $loc_{bnext\_i}$ is the expected next position of egret B.

Egret C tends to actively pursue prey, which adopts an encircling mechanism for its position update. The encircling mechanism is calculated by

(16) $$D_{h}=loc_{selfbest\_i}\;-loc_{self\_i},$$

(17) $$D_{g}\;=loc_{selfbest\_g}-loc_{self\_g},$$

(18) $$loc_{cnext\_i}=(1-t_{i}\;-t_{g}\;)\cdot loc_{self\_i}+t_{h}\;\cdot D_{h}\;+t_{g}\;\cdot D_{g},$$

(19) $$fit_{cnext\_i}\;=f(loc_{cnext\_i}),$$

where *D_h_* is the gap matrix between the current position of the egret group and the best position, *D_g_* is the gap matrix between the optimal positions of all egret squads, and $loc_{cnext\_i}$ represents the expected position of egret *C*. *t_h_* and *t_g_* are random numbers in the range [0, 0.5).

(3) Discriminant Condition

Each member of the egret group has decided on their own plan; the group selects the optimal strategy and takes action together. The specific process is as follows:

(20) $$loc_{snext\_i\;}\;=[loc_{anext\_i\;},loc_{bnext},loc_{cnext\_i\;}\;],\;$$

(21) $$\;fit_{snext\_i\;}\;=[fit_{anext\_i\;},fit_{bnext},fit_{cnext\_i\;}\;],$$

(22) $$\;c_{i}\;=argmin(fit_{snext\_i\;}),$$

(23) $$loc_{self\_i}=\left\{\begin{array}{l} loc_{sself\_i}{|}_{c_{i}}\;if\;loc_{sgoal\_i}{|}_{c_{i}}<loc_{goal\_i}\;or\;r<0.3,\\ loc_{self\_i}\;\;\;\;else, \end{array}\right.$$

where $loc_{snext\_i\;}$ is the solution matrix of the first white egret class.

### 2.2. Human Evolutionary Optimization Algorithm

The human evolutionary optimization algorithm (HEOA) is inspired by the human evolutionary process; the adaptation and exploration behaviors of early humans are adopted to explore new environments and resources [26]. This algorithm simulates the exploration and development stages of human evolution, which can search for solutions to optimization issues.

(1) The Stage of Human Exploration

At this stage, the algorithm extensively searches the solution space to discover potential solutions. It is similar to the early exploration stage of human civilization: humans explore unknown territories in search of new resources and opportunities. The specific process is as follows:

(24) $$\begin{array}{l} loc_{i}^{\left(t+1\right)}=\beta \cdot (1-\frac{t}{Max_{iter}})\cdot (loc_{i}^{t}-loc_{best})\cdot Levy(dim)\\ \;\;\;+loc_{best}\cdot (1-\frac{t}{Max_{iter}})+(loc_{mean}^{t}-loc_{best})\cdot floor(\frac{rand}{f_{jump}})\cdot f_{jump}, \end{array}$$

where β is the adaptive parameter. The optimal performance value for β in experiments was found to be 0.2, meaning the perturbation amplitude of the Lévy distribution performs best when controlled at this level. Therefore, in subsequent experiments, this parameter value was uniformly set to 0.2 throughout this study. t represents the current iteration, dim denotes the dimension of the problem or the number of involved variables, rand is a random number in the range [0, 1], $loc_{i}^{t}$ is the current position, $loc_{i}^{\left(t+1\right)}$ represents the subsequent updated position, $loc_{best}$ is the global optimal solution obtained by HEOA, Levy represents the heavy-tailed distribution, and jump is the jumping strategy.

(2) The Stage of Human Development

Potential solutions are discovered, then the algorithm focuses on optimizing and improving these solutions. This phase is similar to the development stage of human civilization: humans continuously improve and optimize existing technologies and knowledge to enhance efficiency and quality. The specific process is as follows:

(25) $$loc_{i}^{t+1}=R_{n}\cdot \exp(\frac{loc_{worst}^{t^{2}}-loc_{i}^{t^{2}}}{i^{2}}),$$

where $loc_{worst}^{t^{2}}$ represents the position of the least adapted individual in the population at iteration t2, and Rn represents a random number following a normal distribution.

In the human development stage, the population is classified into leaders, explorers, followers, and losers, and they conduct different updating strategies. The strategy of a leader is written as

(26) $$loc_{i}^{t+1}=\left\{\begin{array}{c} \omega \cdot loc_{i}^{t}\cdot exp(\;\frac{-t}{rand\cdot Max_{iter}}\;),R<\Omega ,\\ \omega \cdot loc_{i}^{t}+R_{n}\cdot ones(1,dim),R\geq \Omega , \end{array}\right.\;$$

where ones(1,dim) is a function that generates a row vector containing dim, R is a random number, Ω represents the evaluation value, and ω represents the coefficient of ease for knowledge acquisition. Moreover, the explorer strategy is computed by

(27) $$loc_{i}^{t+1}=R_{n}\cdot exp(\frac{loc_{worst}^{t^{2}}-loc_{i}^{t^{2}}}{i^{2}}),$$

where $loc_{worst}^{t^{2}}$ is the position of the individual with the worst fitness in the t-th iteration.

The follower strategy is calculated by:

(28) $$loc_{i}^{t+1}=loc_{i}^{t}+\omega \cdot R_{d}\cdot (loc_{best}^{t}-loc_{i}^{t}),$$

where $loc_{best}^{t}$ represents the position of the individual with the best fitness in the population at t iteration, and Rd is a random number.

The loser strategy is given by

(29) $$loc_{i}^{t+1}=loc_{best}+(loc_{best}-loc_{i}^{t})\cdot R_{n}.$$

### 2.3. Mutation Operation

(1) Gaussian Mutation

To further enhance the search capability and local optimization ability of the ESOA, this paper integrates the Gaussian mutation (GM) operation [23]. It is an optimization method that generates new positions by applying random numbers that comply with a normal distribution to the original position vector. This mutation strategy allows most mutation operators to focus around the original position, thus effectively performing a local search into a small range, which helps the algorithm escape from the local optimum. The Gaussian mutation formula is as follows:

(30) $$Gaussan=\frac{1}{\sqrt{2\pi }\sigma }e^{-\frac{{\left(x-u\right)}^{2}}{2\sigma^{2}}},$$

where u represents the mean or expected value of the distribution and σ is the standard deviation. The ESOA simulates the cooperative foraging behavior of egret swarms. During its random search phase, the algorithm requires broad exploration of the solution space to discover potential prey, i.e., the global optimum. Introducing Gaussian mutation into the search phase of the ESOA can enhance the algorithm’s global exploration capability in the early iterations, preventing premature convergence due to swarm behavior and avoiding local optima.

The corresponding biological logic can be explained as follows: when egrets randomly search for prey in unknown waters, their movement trajectories exhibit jumpiness and suddenness, which aligns with the long-tailed, leaping characteristics of Gaussian mutation.

(2) Cauchy Mutation

Based on the local optimization performance of the HEOA, this paper adopts the Cauchy mutation operation to further improve its global search capability [25]. It generates new candidate solutions by randomly perturbing the solution vector through the introduction of random numbers from a heavy-tailed distribution. Moreover, the Cauchy density function is as follows:

(31) $$f(x;x_{0},r)=\frac{1}{\pi \gamma [1+{\left(\frac{x-x_{0}}{\gamma }\right)}^{2}]}=\frac{1}{\pi }[\frac{\gamma }{{\left(x-x_{0}\right)}^{2}+\gamma^{2}}],$$

where x represents the value of the random variable, $x_{0}$ represents the location parameter, γ is the scale parameter, and π represents pi. The HEOA simulates knowledge inheritance and gradual innovation in human society. This algorithm emphasizes stable, incremental optimization based on existing experience. Introducing Gaussian mutation into the local refinement phase of the HEOA can enhance the algorithm’s fine-grained exploitation capability of the solution space in later iterations, improving convergence accuracy and stability.

The corresponding behavioral logic can be interpreted as humans often making minor optimizations based on existing solutions in technological improvements, such as process fine-tuning, which is consistent with the local perturbation characteristics of Gaussian mutation.

## 3. E-HEOA

### 3.1. The Prediction Task Process of the E-HEOA

**Figure 2:**
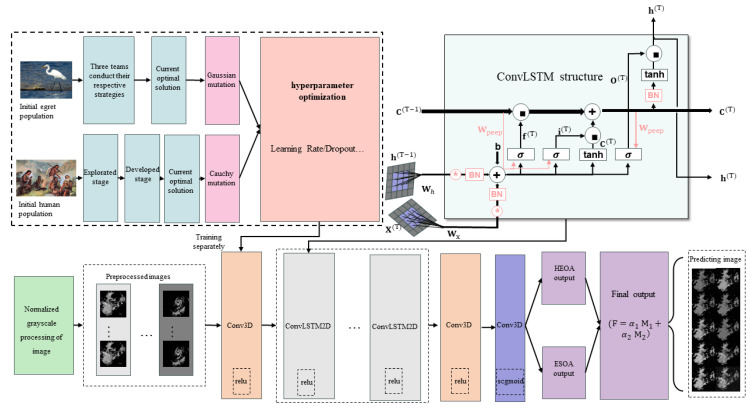
The prediction task process of the E-HEOA.

The prediction task process of the E-HEOA is shown in Figure 2. In addition, the associated optimization algorithm conducts the prediction task in three major steps: data preprocessing and input, E-HEOA training, and E-HEOA execution for the prediction task and output of results.

Firstly, this study conducts data preprocessing on the raw radar echo images obtained from the radar transmission signal. Specifically, we perform operations such as cropping, scaling, gray-scaling and normalization on the raw images, and then the processed images are divided into batches of 20 frames. After dividing the image data into training and validation sets, they are input into the associated optimization algorithm to carry out subsequent training and prediction tasks.

Secondly, the associated optimization algorithm employs two optimization algorithms to search for the optimal learning rate and dropout rate of the ConvLSTM2D-CNN based on the E-HEOA. When the optimization algorithms achieve the preset number of iterations, the search is complete. Moreover, the final values for the dropout rate and learning rate are obtained.

During the process of performing the prediction task, the forward propagation of the model first passes through two 3D convolutional layers (Conv3D) activated by the Relu activation function. Then, it successively passes through two hidden layers composed of the ConvLSTM2D layer, dropout layer, and batch normalization layer stacked in sequence to conduct the prediction task. After passing through two Conv3D layers, the output layer adopts a Conv3D layer activated by the Sigmoid activation function to perform binary classification on the prediction results.

Lastly, the ConvLSTM2D-CNN is guided by two sets of hyperparameters, which are obtained through adaptive optimization by the ESOA with Gaussian mutation and HEOA with Cauchy mutation, thus producing two sets of prediction results. In addition, they are combined by using weighted summation, then the weight summation formulas are shown in (32) and (33). f is the final prediction after weighted summation, M1 is the training result based on the ESOA, α1 represents the weight assigned to the ESOA, M2 is the training result based on the HEOA, and α2 represents the weight assigned to the HEOA.

(32) $$f=\alpha_{1}\cdot M_{1}+\alpha_{2}\cdot M_{2},$$

(33) $$\alpha_{1}+\alpha_{2}=1.$$

Specifically, the joint method used in the proposed E-HEOA model refers to the fusion of prediction results. That is, it combines the prediction outputs from the ConvLSTM2D-CNN tuned via the Cauchy mutation-improved ESOA and those from the ConvLSTM2D-CNN tuned via the Gaussian mutation-improved HEOA, achieving pixel-level fusion of the predicted result images. This fusion approach offers strong intuitiveness, adjustability, and scalability.

### 3.2. The Associated Optimization Algorithm for the E-HEOA

(1) Hyperparameter Selection and Population Initialization for the Associated Framework

**Figure 3:**
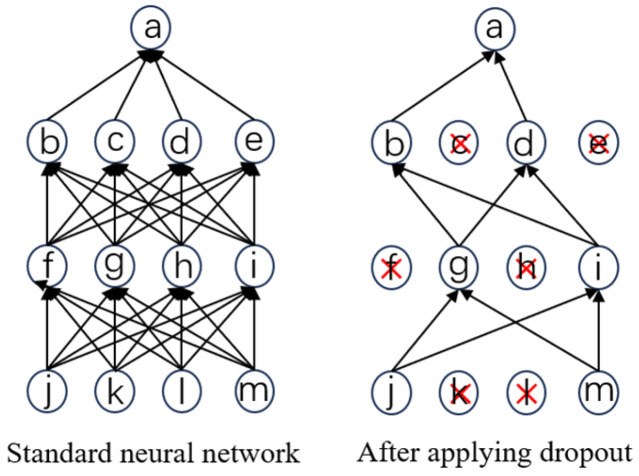
The E-HEOA conducts the dropout process.

In the E-HEOA, the associated algorithm optimizes two hyperparameters: the dropout rate and the learning rate. When dropout is performed in the associated optimization algorithm, a portion of connections of the network is randomly disconnected during each iteration based on the set dropout rate, which is illustrated in Figure 3. This process reduces the possibility of co-adaptation to noise from previous layers, thereby making the model more robust. By doing so, the network is prevented from overly relying on certain features, which in turn reduces the risk of model overfitting.

The learning rate output by the associated optimization algorithm influences the update rate of the network parameters’ weights during the model training process. In the training of the associated optimization model, the learning rate guides the adjustment of network weights, which is shown in Equation (34). θt represents the old weight, θt + 1 represents the new weight, η represents the learning rate, and $\partial J/\partial \theta_{t}$ represents the gradient. An appropriate learning rate can enable the objective function to converge into a local minimum within a suitable timeframe.

(34) $$\theta_{t+1}=\theta_{t}+\eta \cdot \frac{\partial J}{\partial \theta_{t}}.$$

(2) The Fitness Function

The fitness of the associated optimization algorithm in the E-HEOA model is calculated by the fitness function, which is as follows:

(35) $$x_{fit\_\min\_turn}=\min(x_{loss_{\min_{1}}},x_{loss_{\min_{2}}}\ldots x_{loss_{\min_{i}}})\;(i\in N),$$

(36) $$x_{fit\_best}=x_{loss\_\min\_turn},$$

where $x_{loss\_\min}$ is the minimum loss value for this individual at the current iteration, $x_{loss\_history}$ is the loss value record array for this individual at the current iteration, *i* is the number of loss values in the loss value record array, N is a natural number, and $x_{fit\_best}$ defines the optimal fitness.

Thereafter, the fitness function compares the optimal fitness of each individual in the entire population to obtain the current optimal fitness for the entire population in this iteration. In addition, the current optimal fitness is compared with the global optimal fitness. If the current optimal fitness is higher than the global optimal fitness, the current optimal fitness value is assigned to the global optimal fitness. If the current optimal fitness is less than the global optimal fitness, it is not assigned. The mathematical model is shown in Equations (37) and (38). $x_{fit\_best\_turn}$ is the current optimal fitness at the current iteration, $x_{fit\_best\_all}$ is the global optimal fitness, j is the number of individuals in the population, and N is a natural number.

(37) $$x_{fit\_best\_turn}=\max(x_{fit\_best_{1}},x_{fit\_best_{2}}\ldots x_{fit\_best_{j}})\;(j\in N),$$

(38) $$x_{fit\_best\_all}=\left\{\begin{array}{l} x_{fit\_best\_all}=x_{fit\_best\_turn}\;if\;x_{fit\_best\_all}<x_{fit\_best\_turn},\\ x_{fit\_best\_all}\;\;\;\;\;\;\;else. \end{array}\right.$$

(3) The Updating Process of Population Location

After the global optimal fitness update is completed in this iteration, the egret swarm optimization algorithm and the human evolutionary optimization algorithm respectively update the positions of their populations. They can pass new positions to the fitness function in the next iteration for hyperparameter optimization [47,48,49,50,51,52,53,54,55].

(a) The ESOA Based on Gaussian Mutation

The ESOA based on Gaussian mutation consists of three egrets: egret A adopts the guided forward mechanism, while egret B and egret C adopt the random walk mechanism and the encircling mechanism, respectively. Egret A estimates the gradient of the plane parameters to descend the plane, thus performing a search. Egret B performs global random wandering, and egret C conducts selective exploration based on the position of a better egret. Moreover, the ESOA based on Gaussian mutation achieves a better balance between development and exploration and can perform a quick search for feasible solutions. The pseudocode for the ESOA based on Gaussian mutation is shown in Algorithm 1.

(b) The HEOA Based on Cauchy Mutation

The HEOA based on Cauchy mutation divides the global search process into two stages: the human exploration stage and the human development stage. In the human exploration stage, the fitness is calculated, and then the search space is explored based on the fitness value. In the human development stage, the population is classified into leaders, explorers, followers and failures; each category adopts different search strategies. In particular, the pseudocode for the HEOA based on Cauchy mutation is shown in Algorithm 2.
**Algorithm 1:** ESOA based on Gaussian Mutation.**Input:** $loc_{self\_i}$, $step_{a}$, $step_{b}$**Operation**/* *Sit-and-wait* step */1.**Update** the integrated gradient ***g*** via Equations (4)–(8)2.**Update** the weight of observation method ***w*** by Equations (9)–(11)3.**Get** the expected position $loc_{anext\_i}$ of Egret A by Equation (13)4.**Retrieve** the Egret A’s fitness $fit_{anext\_i}$
5.**Return** $loc_{anext\_i}$, $fit_{anext\_i}$/* *Aggressive* step */6.**Get** the expected position $loc_{bnext\_i}$ of egret B by Equations (14) and (15)7.**Get** the expected position $loc_{cnext\_i}$ of egret C by Equations (18) and (19)8.**Retrieve** the fitness of egret B $fit_{bnext\_i}$ and egret C $fit_{cnext\_i}$
9.**Return** $loc_{bnext\_i}$, $loc_{cnext\_i}$, $fit_{bnext\_i}$, $fit_{cnext\_i}$/* *Search the global optimum based on Gaussian mutation*-step */10.   **while**
$t<t_{\max}$
11.    **Implement** *Sit-and-wait step*, **Obtain**
$loc_{anext\_i}^{\;t}$, $fit_{anext\_i}^{\;t}$
12.    **Implement** *Aggressive step*, **Obtain**
$loc_{bnext\_i}^{\;t}$, $fit_{bnext\_i}^{\;t}$, $loc_{cnext\_i}^{\;t}$, $fit_{cnext\_i}^{\;t}$
13.    **Get** next position via Equations (20)–(23)
14.    $loc_{best}=Gauss(loc_{self\_i}^{\;t+1},loc_{best})$
15.    **Retrieve** the fitness of best position $fit_{best}$
16.    **Return**
$loc_{best}$, $fit_{best}$
/* Operation Ending */**Output:** $loc_{best}$, $fit_{best}$

**Algorithm 2:** HEOA based on Cauchy Mutation.**Input:** 
$loc_{i}^{t}$

**Operation**
/* *Human exploration* step */1.**Get** the current position by Equation (24)2.**Return** $loc_{i}^{\left(t+1\right)}$/* *Human development* step */6./*Leader*/: **Get** the current position by Equation (26)7./*Explorer*/: **Get** the current position by Equation (27)8./*Follower*/: **Get** the current position by Equation (28)9./*Loser*/: **Get** the current position by Equation (29)/* *search the global optimum based on Cauchy mutation* step */10.  **while**
$t<t_{\max}$
11.   **Implement** *Human exploration step*, **Obtain**
$loc_{i}^{\left(t+1\right)}$
12.   **Implement** *Human development step*, **Obtain**
$loc_{i}^{\left(t+1\right)}$
13.   $loc_{best}=Cauthy(loc_{i}^{t+1},loc_{best})$
14.   **Retrieve** the fitness of best position $fit_{best}$
15.  **Return**
$loc_{best}$, $fit_{best}$
/* Operation Ending */**Output:** $loc_{best}$, $fit_{best}$

(c) The ConvLSTM2D-CNN Model for the E-HEOA

**Figure 4:**
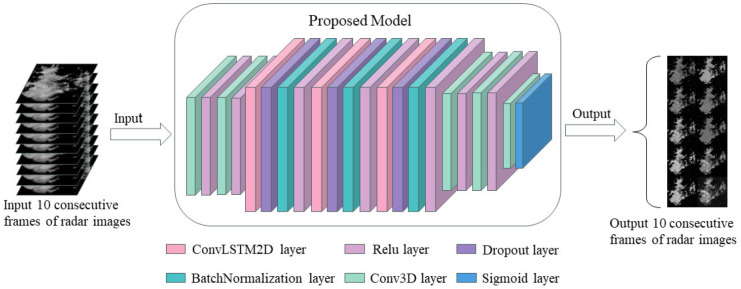
The ConvLSTM2D-CNN model.

The hierarchical structure of the ConvLSTM2D-CNN model in the E-HEOA is shown in Figure 4. It consists of two 3D convolutional layers (Conv3D) activated by the Relu activation function. Thereafter, there are two hidden layers stacked in sequence, which are composed of ConvLSTM2D layers, dropout layers and batch normalization layers. Two Conv3D layers are activated by the Relu activation function before the output. Furthermore, the output layer adopts a Conv3D layer activated by the Sigmoid activation function for binary classification of the prediction results.

## 4. Experimental Results Analysis

### 4.1. The Dataset and Related Work

(1) Dataset

The dataset is derived from the publicly available HKO-7 radar echo image dataset [25]. Each radar image covers the 101 km × 101 km area around the target location, capturing radar reflectivity. The radar images are measured at fifteen different time intervals with a six-minute gap; they have four different altitudes ranging from 0.5 km to 3.5 km, which also achieves a 1 km interval. The total time span of the dataset is two years, which obtains a total of 170,000 images.

(2) Data Preprocessing

Data preprocessing is performed to make the data suitable for model training and evaluation. In the preprocessing process of this paper, the following steps are carried out:

Step 1: Image Loading. Radar image data is loaded from the original dataset. Each radar image is initially in grayscale; the grayscale radar images are converted into numerical representations to facilitate data processing.

Step 2: Image Resizing. To ensure image input with the same dimensions, the images are resized to the same width and height, thus matching the input size requirements of the model.

Step 3: Data Normalization. To accelerate the model’s convergence and prevent issues like gradient explosion or vanishing gradients, the image data is normalized. The pixel values are scaled to a range between 0 and 1, thereby making it easier for the model to process this data.

**Figure 5:**
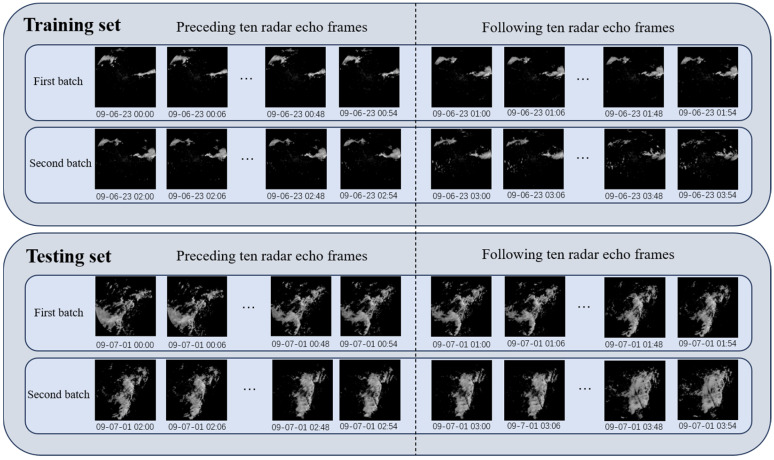
The prediction process for training and testing sets.

Based on these preprocessing steps, a dataset suitable for the model input is obtained. The dataset is split into a training set and a test set in a 4:1 ratio. During the experiments, batches of the preceding ten radar echo frames are input into the associated optimization model to predict the following ten frames. Moreover, the effectiveness of the proposed prediction method is validated by comparing the results between the training and test sets, which is illustrated in Figure 5.

### 4.2. Experimental Approach

**Figure 6:**
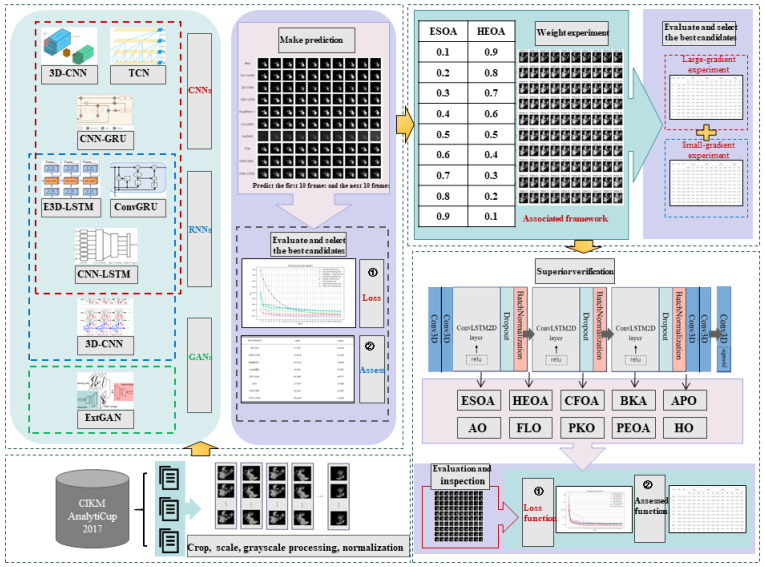
The major process of the experimental setup.

The major research approach of this paper can be summarized in the following three steps: selecting a neural network with strong image prediction performance, determining the optimal weights for each algorithm with an associated learning framework and comparing the prediction results of the E-HEOA method with the ConvLSTM2D-CNN optimized by other hyperparameter search algorithms. The major experimental setup is shown in Figure 6.

The first step of this research is to select a neural network with excellent image prediction capabilities. This step aims to choose a neural network that already has decent image prediction performance before hyperparameter tuning, then we verify the superiority of the neural network after optimization.

The second step is to find the optimal weights of various algorithms with the associated learning framework. In this step, the optimal weight allocation for the ESOA and HEOA is determined with the associated learning framework. The goal is to combine the advantages of the two algorithms and mitigate their individual shortcomings by their optimized prediction results, thus achieving a prediction performance that is better than a single optimization algorithm.

The final step is to compare the prediction results of the E-HEOA that underwent hyperparameter tuning based on other algorithms. This aims to validate the superiority of the E-HEOA in completing radar image extrapolation tasks through a comparison with the baseline algorithms.

### 4.3. The Selection of the Neural Network

(1) Benchmark Models

This paper compares eleven mainstream image prediction models, including the three-dimensional convolutional neural network (3D-CNN) [28], three-dimensional long short-term memory network (E3D-LSTM) [29], enhanced predictive recurrent neural network (PredRNN++) [30], convolutional gated recurrent unit (ConvGRU) [31], extended generative adversarial network (ExtGAN) [9], temporal convolutional network (TCN) [32], convolutional neural network–gated recurrent unit (CNN-GRU) [33], convolutional neural network–long short-term memory network (CNN-LSTM) [34], Memory in Memory (MIM) [35], Trajectory GRU (TrajGRU) [36] and Earthformer [37]. These models cover most of the neural networks used for image prediction tasks; each model has excellent strengths and performance characteristics.

(a) 3D-CNN: It is an extended form of a CNN with convolution kernel slides in three-dimensional space (height, width, and depth), which can capture both spatial and temporal features of the image.

(b) E3D-LSTM: It combines the advantages of 3D convolution and LSTM by embedding 3D convolution layers into LSTM, thus enhancing the capability of the model to capture spatiotemporal dynamics.

(c) PredRNN++: It is a recurrent neural network designed for sequence prediction tasks like video prediction, which integrates hierarchical memory units and bidirectional interaction mechanisms to address gradient vanishing or explosion issues in traditional RNNs.

(d) ConvGRU: It is a convolutional version of GRU; the fully connected layers of the traditional GRU are replaced by convolutional layers to process image data.

(e) ExtGAN: It extends the traditional GAN by concatenating the input data with the generated data from the generator during training, which forms new inputs for the discriminator.

(f) TCN: It is a convolutional neural network, which is designed to handle time-series data. It also adopts dilated convolutions to capture long-term dependencies, thus offering higher parallelism and a shorter training time than traditional RNNs.

(g) CNN-GRU: It combines a CNN and GRU, then the CNN extracts spatial features from the input data and the GRU performs sequence modeling which is suitable for tasks that require handling both spatial and temporal data.

(h) CNN-LSTM: It is similar to CNN-GRU, but it replaces GRU with LSTM, which has a more complex gating mechanism to handle long-term dependencies.

(i) MIM: It is a recurrent neural network variant designed for modeling long-term dependencies in time series. Its core innovation lies in a cascaded recurrent memory module that explicitly captures and propagates complex temporal trends across multiple time steps.

(j) TrajGRU: It is an improved version of ConvGRU, which addresses the inaccuracy of optical flow estimation by using learnable local sampling positions to dynamically adjust the receptive field of convolutional kernels, thereby modeling the motion of cloud clusters and other objects more precisely.

(k) Earthformer: It is a general-purpose spatiotemporal prediction Transformer architecture for Earth system science. Its core mechanism is a cube attention module, which treats spatiotemporal data as a 3D cube for efficient modeling, balancing computational complexity and the ability to capture long-range dependencies.

(2) Parameter Setting

In multiple repeated experiments, we find that the hyperparameters are set to a learning rate of 0.001 and a dropout rate of 0.2. These selected models and the ConvLSTM2D-CNN model based on the 3D-CNN model obtain excellent performance. The purpose of this model selection step is to manually select the model with the best prediction performance for further optimization without automating hyperparameter selection. This verifies that the optimized model performs better on most evaluation metrics, and the hyperparameters of the selected models and the ConvLSTM2D-CNN are uniformly set manually to a learning rate of 0.001 and dropout rate of 0.2. Additionally, the batch size of the input image data, the number of epochs and the image size also directly affect the prediction results of the neural network. To ensure the validity of the experimental comparison results, these input parameters are also uniformly controlled. Moreover, during the experimental process, the number of iterations for the evolutionary algorithm is set to 50, and the population size is set to 100. The parameter settings for this experimental step are shown in Table 1.

(3) Model Evaluation Method and Analysis of Prediction Results

This paper conducts a quantitative evaluation of neural networks from two perspectives: firstly, the evaluation of the model’s prediction results and secondly, the evaluation of the model’s convergence speed. Specifically, the paper evaluates the prediction results and convergence speed of eight mainstream models, and then we select the best model among them, thus optimizing and improving this model. Finally, the eight mainstream models are adopted as the benchmark to validate the superiority of the proposed model.

(a) Model Evaluation Metric

Evaluation Function: To reflect the performance of the prediction results of the neural networks, this paper adopts two evaluation functions to assess and analyze the prediction results: Mean Square Error (MSE) [38] and Structural Similarity Index Measure (SSIM) [39]. MSE is written as follows:

(39) $$MSE=\frac{1}{n}\sum_{i=1}^{n}{\left(y_{i}-{\hat{y}}_{i}\right)}^{2},$$

where *n* is the number of the samples, *y_i_* is the true value, and ${\hat{y}}_{i}$ is the predicted value.

SSIM is a relatively comprehensive evaluation metric which breaks down the comparison of the similarity between the ground truth image and the predicted image into three dimensions: luminance, contrast, and structure. The similarity between the ground truth image and the predicted image is a function of these three components. SSIM is computed by

(40) SSIM(x,y)=f(l(x,y),c(x,y),s(x,y)),

where *x* is the truth image, *y* is the predicted image, *l*(*x*, *y*) is the luminance similarity, *c*(*x*, *y*) is the contrast similarity, and *s*(*x*, *y*) is the structure similarity.

If an image has *N* pixels and the pixel value of each pixel is *x_i_*, the average luminance of the image *u_x_* is expressed in (41). The luminance similarity is represented in (42); *C*_1_ can prevent the denominator in (42) from being zero.

(41) $$\mu_{x}=\frac{1}{N}\sum_{i=1}^{N}x_{i},$$

(42) $$l(x,y)=\frac{2\mu_{x}\mu_{y}+C_{1}}{\mu_{x}^{2}+\mu_{y}^{2}+C_{1}}.$$

Contrast reflects the intensity of variation in brightness for an image; *σ_x_* represents the standard deviation of the pixel values, which is shown in Equation (43). Moreover, the contrast similarity is written in (44), and *C*_2_ can prevent the denominator in (44) from being zero.

(43) $$\sigma_{x}={\left(\frac{1}{N-1}\sum_{i=1}^{N}{\left(x_{i}-\mu_{x}\right)}^{2}\right)}^{1/2},$$

(44) $$c(x,y)=\frac{2\sigma_{x}\sigma_{y}+C_{2}}{\sigma_{x}^{2}+\sigma_{y}^{2}+C_{2}}.$$

Structure cannot be represented by a scalar; it should be represented by a vector consisting of all the pixels in the image. The structure similarity is expressed in Equation (46). *σ_xy_* is the covariance between *σ_x_* and *σ_y_*, which is defined in (45); *C*_3_ can prevent the denominator in (46) from being zero.

(45) $$\sigma_{xy}=\frac{1}{N-1}\sum_{i=1}^{N}\left(x_{i}-\mu_{x})(y_{i}-\mu_{y}\right),$$

(46) $$s(x,y)=\frac{\sigma_{xy}+C_{3}}{\sigma_{x}\sigma_{y}+C_{3}}.$$

The mean and variance frequently fluctuate significantly across the span of the entire image. The SSIM evaluation method adopts a sliding window with a stride of 1 to calculate its corresponding patches in the two images under each sliding window, and then the average is taken as the SSIM of the two images. In addition, SSIM is written as

(47) $$SSIM(X,Y)=\frac{\left(2\mu_{x}\mu_{y}+C_{1})(2\sigma_{xy}+C_{2}\right)}{\left(\mu_{x}^{2}+\mu_{y}^{2}+C_{1})(\sigma_{x}^{2}+\sigma_{y}^{2}+C_{2}\right)},$$

(48) $$SSIM=\frac{1}{M}\sum_{j=1}^{M}SSIM(x_{i},y_{i}).$$

Briefly, MSE is a common statistical metric, used to analyze the performance of prediction models, and SSIM is a frequently used evaluation metric in the field of image prediction. Specifically, MSE measures the gap between the predicted values and the true values; the closer the MSE is to 0, the better the model performance. In the field of radar extrapolation, an MSE below 40 can be considered a good prediction result. By considering the brightness, contrast, and structural information of the image, SSIM can reflect whether the restored image has preserved the structural information of the original image by comparing it with other metrics. It evaluates image quality by measuring structural similarity. An SSIM above 0.7 can be considered a good prediction result. The evaluation results of various models are shown in Table 2.

Specifically, Table 2 shows that the 3D-CNN model achieves the optimal MSE value, while the TCN ranks second in MSE, and the TCN model attains the optimal SSIM value, with PredRNN++ ranking second in SSIM. Through this comparison, it can be concluded that the 3D-CNN and TCN are the two models with the best overall performance in terms of prediction accuracy.

(b) Loss Function

Binary Cross-Entropy is a commonly used loss function in machine learning [40], especially for binary classification tasks. It is a special case of the cross-entropy loss function applied to binary classification issues, which can be calculated by

(49) $$L(y,\hat{y})=-\frac{1}{N}\sum_{i=1}^{N}\left[y_{i}\log({\hat{y}}_{i})+(1-y_{i})\log(1-{\hat{y}}_{i})\right],$$

where *L* represents the loss function, *N* is the number of samples, *y_i_* is the true label of the *i*-th sample, and ${\hat{y}}_{i}$ is the predicted probability of the *i*-th sample.

(c) Evaluation Results and Analysis

The Selection of Basic Models: Predicted image evaluation results: According to the MSE and SSIM evaluation results in Table 2, it can be observed that the ranking of the models based on the MSE evaluation from smallest to largest is 3D-CNN, TCN, Earthformer, MIM, PredRNN++, TrajGRU, ConvGRU, E3D-LSTM, CNN-GRU, CNN-LSTM, and EXT-GAN. Meanwhile, the ranking based on the SSIM evaluation from largest to smallest is TCN, PredRNN++, MIM, 3D-CNN, ConvGRU, E3D-LSTM, CNN-GRU, CNN-LSTM, TrajGRU, Earthformer and EXT-GAN. In particular, it can be seen that the 3D-CNN and TCN are the two neural networks with the best results by the two evaluation methods, respectively.

**Figure 7:**
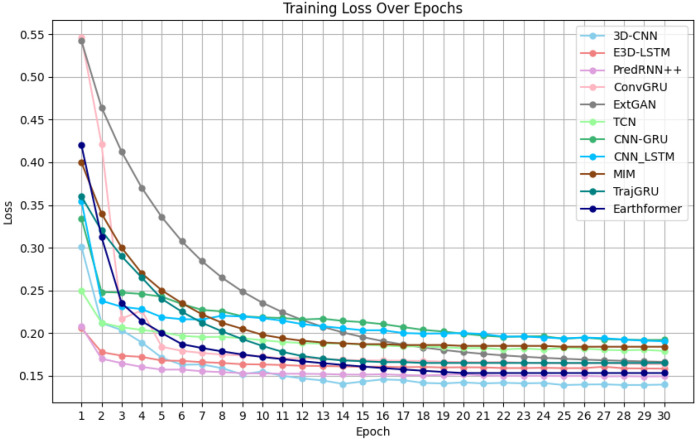
The loss function curves of the training set for eleven advanced models.

**Figure 8:**
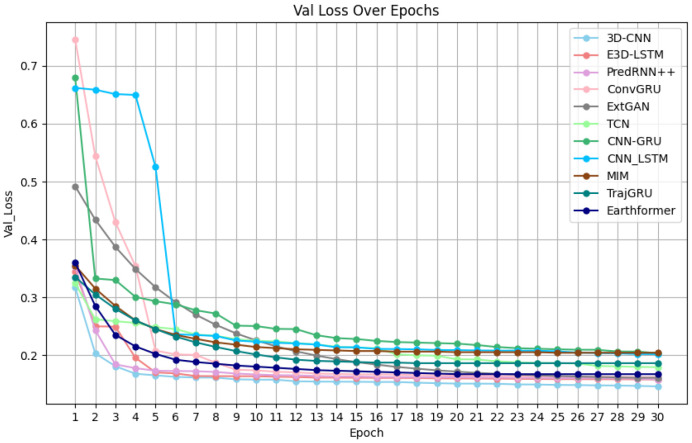
The loss function curves of the testing set for eleven advanced models.

Model performance: Based on the loss function curves of the training set for the eight mainstream models, which are shown in Figure 7, it can be observed that the eight models begin to converge around the 20th iteration. The final loss value at the 30th iteration reflects the fitting performance of the models in the following order from best to worst: 3D-CNN, PredRNN++, Earthformer, E3D-LSTM, ConvGRU, EXT-GAN, TrajGRU, TCN, MIM, CNN-LSTM and CNN-GRU. Additionally, from the loss function curves of the validation set in Figure 8, it can be seen that E3D-LSTM, 3D-CNN, PredRNN++, ConvGRU, EXT-GAN, TCN, CNN-LSTM, CNN-GRU, MIM, TrajGRU, and Earthformer begin to converge at the 7th, 9th, 9th, 14th, 25th, 25th, 25th, 25th, 25th, 25th, and 25th iteration, respectively. The final loss values at the 30th iteration in the validation set reflect the same ranking as the training set: 3D-CNN, PredRNN++, Earthformer, E3D-LSTM, ConvGRU, EXT-GAN, TCN, TrajGRU, CNN-LSTM, MIM and CNN-GRU. Thus, it can be concluded that the 3D-CNN shows the best-fitting performance on both the training and validation sets. Although the convergence speed of the 3D-CNN on the validation set is slightly slower than that of the E3D-LSTM, their convergence speeds on the training set are quite similar.

Comprehensive analysis of various metrics: Based on the evaluation results of the predicted images of eight mainstream models, the models’ convergence speed, and fitting performance on the training and validation sets, this paper finds that the prediction results of the 3D-CNN and TCN obtain optimal performance by two evaluation functions. Additionally, the 3D-CNN demonstrates the best convergence speed and fitting performance on both the training and validation sets among the eight models. Therefore, this paper incorporates the idea of the TCN’s convolution operation and the unique network structure of the 3D-CNN, thus adding the ConvLSTM2D layer into the 3D-CNN model, which forms a new model called ConvLSTM2D-CNN, which can handle time-series data and perform convolution operations at each iteration.

The Validation of the Superiority of the New Model:

Predicted image evaluation results: The evaluation results based on MSE and SSIM in Table 3 show that the order of MSE results from smallest to largest is ConvLSTM2D-CNN, 3D-CNN, TCN, Earthformer, MIM, PredRNN++, TrajGRU, ConvGRU, E3D-LSTM, CNN-GRU, CNN-LSTM, and EXT-GAN. Meanwhile, the order of SSIM results from largest to smallest is ConvLSTM2D-CNN, TCN, PredRNN++, MIM, 3D-CNN, ConvGRU, E3D-LSTM, CNN-GRU, CNN-LSTM, TrajGRU, Earthformer and EXT-GAN. In particular, the new model, ConvLSTM2D-CNN, achieves the best results in MSE and SSIM.

**Figure 9:**
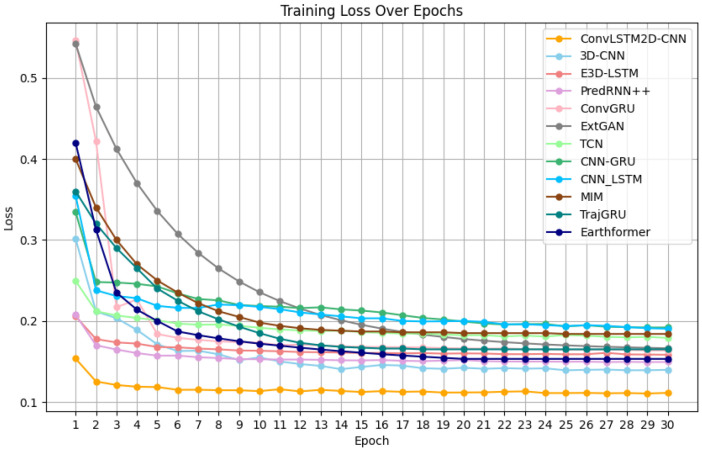
The loss function curves of the training set for eleven advanced models and ConvLSTM2D-CNN.

**Figure 10:**
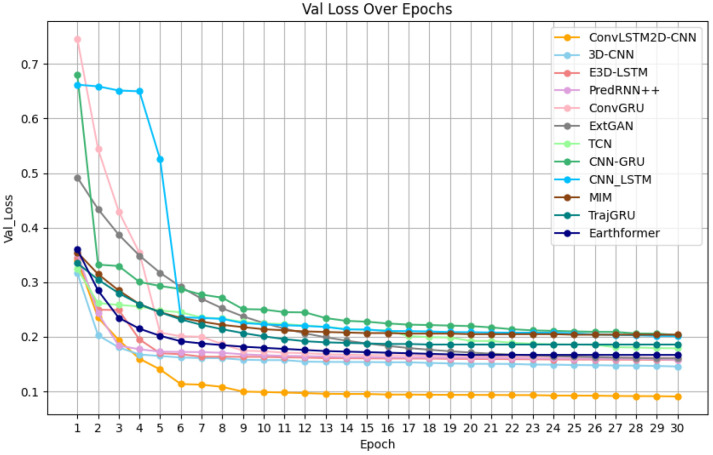
The loss function curves of the testing set for eleven advanced models and ConvLSTM2D-CNN.

Model performance: Based on the loss function curves of eight mainstream models and the new model in the training set, which is shown in Figure 9, it can be observed that the eight mainstream models start to converge around the 20th iteration, while the new model ConvLSTM2D-CNN starts to converge at the 16th iteration. The final loss values at the 30th iteration reflect the fitting performance of the models ranked from best to worst as follows: ConvLSTM2D-CNN, 3D-CNN, PredRNN++, Earthformer, E3D-LSTM, ConvGRU, EXT-GAN, TrajGRU, TCN, MIM, CNN-LSTM and CNN-GRU. Additionally, according to the loss function curves of the eight mainstream models and the new model in the validation set, which is shown in Figure 10, E3D-LSTM starts to converge at the 7th iteration; 3D-CNN and PredRNN++ start to converge at the 9th iteration; the new model ConvLSTM2D-CNN starts to converge at the 10th iteration; ConvGRU starts to converge at the 14th iteration; and EXT-GAN, TCN, CNN-LSTM, MIM, TrajGRU, Earthformer and CNN-GRU start to converge around the 25th iteration. The final loss values at the 30th iteration in the validation set also reflect the same ranking of fitting performance in the training set, which is ranked as follows: ConvLSTM2D-CNN, 3D-CNN, PredRNN++, Earthformer, E3D-LSTM, ConvGRU, EXT-GAN, TCN, TrajGRU, CNN-LSTM, MIM and CNN-GRU. Thus, it can be concluded that the ConvLSTM2D-CNN achieves the best final-fitting performance in both the training and validation sets. Although the ConvLSTM2D-CNN’s convergence speed is slightly slower than E3D-LSTM, the 3D-CNN, and PredRNN++ in the validation set, it has the fastest convergence speed in the training set.

Comprehensive analysis of various metrics: By synthesizing the prediction results of eight mainstream models and the evaluation results of the new model, the convergence speed and fitting performance on the training and validation sets, this paper finds that the new model, ConvLSTM2D-CNN, achieves the best overall performance in terms of evaluation results, convergence speed, and fitting performance. On the one hand, it obtains the rationality of the network design and improvement strategy of the new ConvLSTM2D-CNN model; on the other hand, it also highlights the superiority of the ConvLSTM2D-CNN model over eight mainstream models, thus covering most types of neural networks in the radar image extrapolation task.

### 4.4. The Associated Algorithm Searching for Optimal Weight Parameters

The adaptive optimization of hyperparameters using an associated framework is a major innovation in this paper. The purpose of applying the associated framework is to combine the advantages of two optimization algorithms to develop a new optimization scheme. Meanwhile, the weight parameters of the two optimization algorithms with the associated framework significantly affect the final optimization performance of the associated algorithm. Therefore, this paper conducts gradient experiments on the algorithm weights to find the optimal parameters. The gradient experiment presents the difference in each group’s parameters. Specifically, the weight parameter group is selected at 0.1, 0.2, 0.3, 0.4, 0.5, 0.6, 0.7, 0.8 and 0.9; its gradient is 0.1. The weight parameter group has values of 0.11, 0.12, 0.13, 0.14, 0.15, 0.16, 0.17, 0.18, and 0.19; the gradient is 0.01.

(1) Evaluation Metrics for Weight Optimization Performance

Except for the commonly used evaluation metrics MSE and SSIM, the evaluation metrics for the optimization performance of the weight parameters in the associated algorithm also contain three metrics, which are frequently used in meteorology for comparing predictive performance [41]: Critical Success Index (CSI), Probability of Detection (POD) and False Alarm Rate (FAR). The high CSI and POD indicate that more radar echo grid points are correctly predicted, and a lower FAR means fewer false alarms. The standard for optimal values are as follows: CSI ≥ 0.7, POD ≥ 0.9, and FAR ≤ 0.1.

In this paper, the radar extrapolation prediction results and the corresponding real-time observations are first binarized, which means that grid cells with values above a threshold are set to 1, and others below the threshold are set to 0. Then, we present the number of hit grids *n_h_* (the predicted value is 1, and the actual value is 1), missed grids nm (the predicted value is 0, and the actual value is 1), and false-alarm grids *n_f_* (the predicted value is 1, and the actual value is 0). In meteorological operations, different intensities of radar echoes correspond to varying levels of attention. Thus, this paper sets multiple thresholds for objective evaluation: 15 dbz, 20 dbz, 30 dbz, and 40 dbz, respectively. Therefore, CSI, POD, and FAR are written as

(50) $$CSI=\frac{n_{h}}{n_{h}+n_{m}+n_{f}},$$

(51) $$FAR=\frac{n_{f}}{n_{h}+n_{f}},$$

(52) $$POD=\frac{n_{h}}{n_{h}+n_{m}}.$$

(2) Evaluation and Analysis of Weight Optimization Performance

Large-gradient experiment with gradient 0.1: The purpose of this experiment with larger gradients and zero gradients is to quickly identify the weight parameter range that can obtain better evaluation results. From Table 4, we can see that with a weight of 0.4, all evaluation metrics maintain an excellent level. Specifically, with a weight ratio of 0.4, the evaluation results for MSE and SSIM are 20.3611 and 0.8092, respectively. While the MSE score is not the best among the different weight ratios. The SSIM is the best among all weights. The POD performs better than other weight parameters at each threshold, while the CSI is only slightly inferior at a threshold of 20 dbz with a value of 0.7299. However, when the weight ratio is 0.3, the CSI can reach 0.7303. Although the FAR is not optimal, it is still at a relatively high level compared to other weight parameters. However, it is found that the MSE with a weight of 0.4 is slightly lower than with the weight of 0.0 (the single ESOA), which indicates that the superiority of the combined algorithm is not fully reflected when the weight is 0. The optimization performance is somewhat inferior to that of the single optimization algorithm.

Therefore, a smaller-gradient experiment is conducted to find a better combination for the associated algorithm. It is discovered that a weight of 0.5 shows the best evaluation results in the range surrounding 0.4. Thus, the experiment is conducted with a smaller gradient of 0.01 between the weights of 0.4 and 0.5, aiming to identify a weight that performs better than the others. Consequently, it outperforms the single optimization algorithm in terms of optimization performance.

Small-gradient experiment with gradient 0.01: Based on the results in Table 5, this paper finds that when the weight is 0.48, the MSE shows higher performance than when the weight is 0.4. The MSE with a weight of 0.4 is 19.9989, while the MSE for the single optimization algorithm in Table 4 is 19.8641. Compared with a single optimization algorithm, the MSE has been reduced to within 0.1, thus ensuring that other evaluation results remain excellent. Additionally, other metrics such as SSIM, POD, CSI, and FAR outperform both other weights and the single optimization algorithm in most cases.

From the perspective of optimization algorithm characteristics, the ESOA is based on the egret swarm cooperation mechanism, and its Gaussian mutation strategy places greater emphasis on global exploration, enabling it to capture sudden and nonlinear changing features in radar echoes, such as the initiation of strong convective systems. As a result, it exhibits better structural integrity and sensitivity to extreme values in predicted images. In contrast, the HEOA follows the paradigm of human knowledge inheritance, and its Cauchy mutation is more adept at local fine-tuning, capable of smoothing noise and maintaining the physical continuity of echo movement, thereby enhancing image stability and detail fidelity. When the weight ratio between the two is 0.48, it implies that the contribution of the HEOA is approximately twice that of the ESOA. This balance precisely corresponds to the dual requirements of radar extrapolation tasks: sufficient global exploration capability to capture the abrupt evolution of complex meteorological systems, which primarily relies on the ESOA, and stronger local optimization capability to suppress distortions caused by data noise and model uncertainties in short-term predictions, which primarily relies on the HEOA. This weight ratio fundamentally reflects that, within the result fusion framework, radar image prediction relies more on temporal continuity smoothing, dominated by the HEOA, while being supplemented by moderate structural innovation exploration, contributed by the ESOA. This achieves an optimal trade-off between stability and sensitivity, which is a typical manifestation of the synergy between physical constraints and data-driven modeling in short-term forecasting.

As discussed, this paper ultimately selects 0.48 as the combined weight for the associated algorithm.

### 4.5. Results and Performance Analysis of E-HEOA

(1) Compared Algorithms

Since this paper has already demonstrated the superiority of the proposed ConvLSTM2D-CNN by comparing it with eight other mainstream models, the baseline models of the neural networks in this section adopt the ConvLSTM2D-CNN. Additionally, ten currently popular evolutionary algorithms are selected to adaptively optimize the hyperparameters of the neural networks, ensuring the rationality of the experimental setup in this paper. The following content provides a detailed description of these algorithms.

(a) CFOA: The catch fish optimization algorithm is a novel metaheuristic algorithm inspired by the fishing process of farmers in ponds [42]. Its vital idea is to achieve maximum fish catch through collaborative efforts with a group.

(b) BKA: The black-winged kite algorithm is inspired by the migration and predatory behaviors of the black-winged kite [43], which incorporates the Cauchy mutation strategy and leader strategy to enhance global search capabilities and convergence speed.

(c) APO: The arctic puffin optimization algorithm is a novel metaheuristic algorithm that simulates the arctic puffin’s aerial flight and underwater foraging behaviors [44]. By a behavior-switching factor, it dynamically transitions between exploration and exploitation phases, thus effectively balancing global search and local development.

(d) PKO: The pied kingfisher optimization algorithm is inspired by the unique hunting behaviors and symbiotic relationships observed in the pied kingfisher, including perch hunting, diving for prey, and fostering symbiosis [45]. These behaviors are translated into mathematical models, thus effectively addressing various optimization challenges in different search spaces.

(e) AO: The artemisinin optimization algorithm is a novel metaheuristic algorithm inspired by the artemisinin treatment process for malaria [46].

(f) FLO: The frilled lizard optimization algorithm simulates the unique hunting behaviors of frilled lizards in their natural habitat. It has a distinct structure and innovative iterative methods, thus exhibiting strong adaptive optimization capabilities [56].

(g) PEOA: The preschool education optimization algorithm is inspired by human activities in the preschool education process. This algorithm provides suitable solutions for optimization problems in repeated processes based on the search abilities of its members [57].

(h) HO: The hippopotamus optimization algorithm is inspired by three prominent behavioral patterns observed in hippopotamus life. It simulates their position updates in rivers or ponds, defense strategies against predators and evasion methods [58].

(i) ESOA: It is inspired by the sit-and-wait strategy of the egret and the attack strategy of the great egret [21].

(j) HEOA: It is inspired by the human evolutionary process [24].

(k) PSO: It is a swarm intelligence optimization algorithm where particles search the solution space by following the current best-performing particles [59].

(l) GA: It iteratively optimizes individuals in the population through operations such as selection, crossover, and mutation [60].

(m) DE: Its core mechanism involves generating new trial vectors by scaling the vector difference between two individuals in the population and adding it to a third individual [61].

(n) E-HEOA: The proposed algorithm integrates the ESOA based on Gaussian mutation with the HEOA based on Cauchy mutation.

(2) The Analysis of Model Prediction Results

As shown in Table 6, through a comprehensive comparison of the hyperparameter tuning effects of various optimization algorithms on the ConvLSTM2D-CNN model, the E-HEOA demonstrates significant advantages across all key metrics. In terms of prediction error, the E-HEOA achieves the lowest mean squared error of 19.9989, not only surpassing the suboptimal APO algorithm which scored 19.8641 but also substantially outperforming the HEOA with 21.6842, ESOA with 20.9176, and all other compared algorithms. Regarding structural fidelity, the E-HEOA ranks first with a structural similarity index of 0.8093, clearly exceeding APO’s 0.8020 and all other models. In meteorological operational metrics, the E-HEOA maintains optimal or near-optimal balance between the critical success index and false alarm rate across all reflectivity thresholds from 15 to 40 dbz, with particularly prominent advantages in medium-to-high thresholds above 20 dbz. Notably, in high-reflectivity scenarios where traditional algorithms generally perform poorly, the E-HEOA sustains stable and reliable prediction performance, whereas algorithms such as CFOA, PKO, and PEOA exhibit sharply increased errors and severely degraded metrics.

From the above results, it can be seen that the various evaluation metrics of the E-HEOA are almost all within the excellent value range in this field. Both the MSE and SSIM of the E-HEOA significantly exceed the recognized excellent value range in this field. Furthermore, except for the metrics that achieve the optimal values, the other metrics are close to the optimal values. In comparison, the comprehensive performance of the E-HEOA is superior to HEOA, CFOA, BKA, PKO, AO, FLO, PEOA, HO, PSO, GA and DE. Although the overall excellent performance of the E-HEOA is not much different from the ESOA and APO, the E-HEOA performs better than the ESOA in terms of MSE, SSIM, POD and CSI. Compared with APO, the E-HEOA is superior in SSIM and CSI, and it also obtains minimal differences in other evaluation metrics. Therefore, the proposed E-HEOA model achieves excellent performance in this field, thus outperforming ten mainstream algorithms.

(3) Ablation Experiment Analysis

Based on the ablation experiment results presented in Table 7, it can be observed that the E-HEOA demonstrates optimal or near-optimal performance across the majority of key evaluation metrics. It exhibits particularly significant advantages in structural similarity index, mean squared error, and meteorological indicators within the medium-to-high reflectivity threshold range of 20 to 40 dbz.

Specifically, regarding prediction accuracy and error assessment metrics, the E-HEOA achieves the lowest mean squared error value of 19.9989 among all compared models. This represents an approximate 1.75% reduction compared to the suboptimal GM-ESOA model, which recorded a mean squared error of 20.3539. Furthermore, the E-HEOA significantly outperforms the original HEOA model with a mean squared error of 21.6842, the original ESOA model with 20.9176, and the Unoptimized model that did not employ any optimization algorithm, which had a mean squared error of 21.5041. These results indicate that the E-HEOA effectively minimizes prediction errors for radar echo intensity while demonstrating the strongest fitting capability. Simultaneously, the E-HEOA attains the highest structural similarity index score of 0.8093, representing an approximate 0.9% improvement over the suboptimal GM-ESOA model, which scored 0.8021, and substantially exceeds all other comparative variants. This confirms that the predicted images generated by the E-HEOA most closely resemble real radar images in terms of structural characteristics, luminance distribution, and contrast preservation, delivering superior visual fidelity.

In the meteorological indicator evaluation stratified by reflectivity thresholds, the E-HEOA exhibits particularly outstanding prediction performance at medium-to-high reflectivity thresholds between 20 and 40 dbz, which constitutes a critical challenge in practical weather forecasting operations. At the low reflectivity threshold of 15 dbz, the E-HEOA demonstrates performance comparable to the optimal model.

The analysis reveals that when no optimization algorithm is applied to the network model, or when the ESOA or HEOA is used individually for optimization, all evaluation metrics remain significantly inferior to those achieved by the E-HEOA. Additionally, the GM-ESOA outperforms the CM-ESOA in both mean squared error and structural similarity index, indicating that the ESOA is better complemented by Gaussian mutation, which represents a localized refinement strategy. Conversely, the CM-HEOA surpasses the GM-HEOA across most meteorological indicators, demonstrating that the HEOA is more effectively paired with Cauchy mutation, corresponding to a global exploration strategy. By integrating the advantages of both approaches and further optimizing through an associated framework, the E-HEOA ultimately exceeds any single mutation combination in core performance metrics. Moreover, while achieving the lowest mean squared error, the E-HEOA maintains a high critical success index, indicating robust generalization capability without compromising forecast effectiveness through excessive optimization of training loss.

(4) The Performance Analysis of the Proposed Model

**Figure 11:**
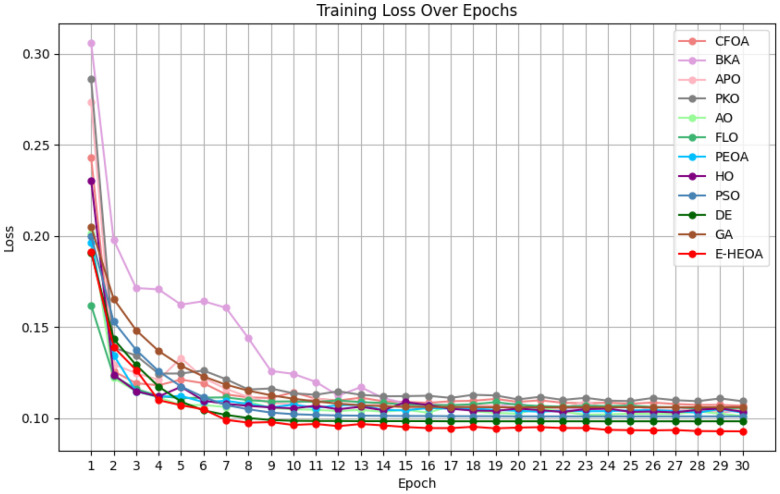
The loss function curves of the training set for ten advanced models and the E-HEOA.

**Figure 12:**
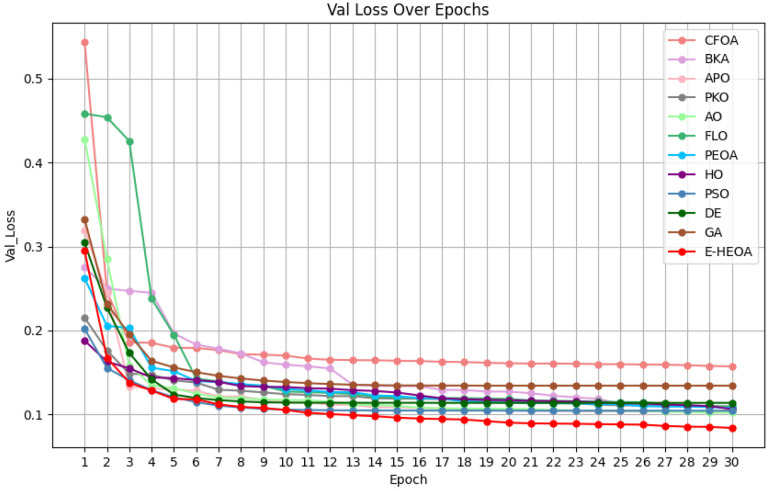
The loss function curves of the testing set for ten advanced models and the E-HEOA.

Note that, since the loss value curve of the E-HEOA is obtained by the weighted summation of the ESOA and HEOA, the loss value curve of the E-HEOA frequently lies between the ESOA and HEOA; thus, it holds no comparative significance. Therefore, the loss function curves of the ESOA and HEOA are not displayed in Figure 11 and Figure 12. From the results in Figure 11, except for BKA converging from the 15th iteration, the other 11 advanced models and the E-HEOA all converge from the 9th iteration on the training set. From the results in Figure 12, except for BKA and APO converging from the 26th and 12th iteration, respectively, the other 11 advanced models and the E-HEOA all converge from the 20th iteration on the validation set. Meanwhile, the final loss value of E-HEOA converges is the lowest on both the training set and validation set. It can be observed that the convergence speed of the E-HEOA is not significantly different from other models; it is also slightly slower than APO on the validation set. The fitting performance of the E-HEOA is optimal on both the training and validation sets. In a word, the proposed E-HEOA model has an excellent convergence speed and fitting performance. In summary, evolutionary algorithms in radar extrapolation optimize model parameters, thus improving prediction accuracy and convergence speed for handling spatiotemporal data.

(5) Analysis of Shorter-Term Prediction Performance

Analysis of the experimental results across different prediction timeframes in Table 8 indicates that the model demonstrates significantly superior performance over shorter prediction intervals, with predictive capability showing a systematic decline as the forecast duration increases. Specifically, under a thirty-minute prediction horizon, the model exhibits optimal overall performance, achieving the lowest MSE value of 19.829 and the highest SSIM value of 0.8148. In terms of meteorological forecast accuracy metrics, using the twenty dbz threshold as an example, the thirty-minute prediction yields a POD of 0.9039, a CSI of 0.7341, and a FAR of 0.2028, all of which outperform the results from sixty-minute and longer prediction intervals. As the forecast duration extends to sixty, ninety, and one hundred and twenty minutes, all key metrics display a consistent degradation trend: MSE progressively rises to 19.9989, 22.47, and 25.08, while SSIM gradually declines to 0.8093, 0.7816, and 0.7524. Meteorological indicators such as CSI also significantly decrease with longer prediction times—for instance, the CSI at the twenty dbz threshold drops from 0.7341 at thirty minutes to 0.6234 at one hundred and twenty minutes—while the FAR correspondingly increases. These data clearly demonstrate that although the model’s prediction accuracy and reliability gradually decline as the forecast time extends, it maintains excellent performance across various short-term prediction intervals overall, highlighting its practical value for nowcasting applications.

(6) Computational Complexity

To characterize the computational cost of our approach, Table 9 presents the worst-case time complexity of the proposed model compared to the individual algorithmic modules that constitute it.

Here, *M* is the population size, and $T_{i}$ is the number of iterations executed by the *i*-th algorithm. The time complexity of an algorithm is primarily determined by the population size and the number of iterations. Therefore, when the population size and the number of iterations are set identically, the time complexity of each algorithm and its mutation-improved variants remains the same.

Specifically, for the E-HEOA, the time complexity may increase due to the use of the joint optimization framework. However, in practice, if a parallel computing approach is employed, allowing the GM-HEOA and CM-ESOA components within the E-HEOA to simultaneously optimize hyperparameters, the time complexity of the joint optimization framework becomes equivalent to that of using a single optimization algorithm for hyperparameter tuning. Moreover, even in the worst-case scenario where parallel computation is not feasible—such as when the GM-HEOA and CM-ESOA components within the joint framework are executed sequentially—the computation time would only increase linearly without causing a qualitative escalation in time complexity.

In this experiment, with the population size uniformly set to 100 and the number of iterations set to 50, the actual time required for prediction typically remains around two hours. Even in the worst case observed across repeated experiments, the actual time consumption has never exceeded three hours. Thus, the proposed E-HEOA model demonstrates favorable performance in terms of both time complexity and computational cost.

(7) Significance Test

Based on the analysis of data from Table 10 and Table 11, the E-HEOA model optimized using the joint optimization framework demonstrates a significant improvement in prediction accuracy compared to the standalone ConvLSTM2D-CNN model. Table 10 shows that across different noise thresholds, the SSIM values of the E-HEOA are consistently higher than those of the ConvLSTM2D-CNN, and the performance gap gradually widens as noise intensity increases, indicating that the E-HEOA exhibits stronger noise resistance. The significance test in Table 11 further supports this conclusion: the E-HEOA achieves a higher average SSIM of 0.8105 compared to ConvLSTM2D-CNN’s 0.8003; with a t-value of 7.19 and a corresponding *p*-value < 0.001, the difference between the two groups is shown to be highly statistically significant. Taken together, the results from both tables indicate that the joint optimization framework not only consistently enhances model accuracy but also significantly improves its robustness.

(8) Sensitivity Analysis of Key Parameters

Based on the systematic parameter sensitivity analysis results presented in Table 12, the influence of population size and iteration count on the prediction performance of the E-HEOA model demonstrates distinct phased characteristics and diminishing marginal returns. In the dimension of population size, when the value increases from 25 to 100, all model metrics show significant linear improvement, with the mean squared error decreasing by 21.7%, the structural similarity index increasing by 11.2%, and the critical success index across various reflectivity thresholds improving by an average of approximately 29.5%. However, once the population size exceeds 100, performance gains markedly slow down; when the population increases from 100 to 125, the mean squared error is further reduced by only 0.54%, indicating that this parameter range has entered a stage of saturation. Regarding the iteration dimension, when the count increases from 50 to 150, the model performance achieves a critical breakthrough, with the mean squared error decreasing by an average of 9.6% under the same population size. Yet, as the iteration count continues to rise to 250, performance gains sharply diminish to less than 0.1%, suggesting that the model has essentially converged after 150 iterations. It is noteworthy that within the parameter space defined by a population size of 100–125 and an iteration count of 150–200, the model attains a global optimal performance plateau. Beyond this range, further increasing parameter configurations not only fails to yield statistically significant performance improvements but also linearly escalates computational costs.

## 5. Conclusions

This study introduces a hybrid optimization algorithm named E-HEOA, designed for radar image extrapolation within a deep learning framework. The approach substantially enhances the quality and precision of radar image predictions by adaptively tuning key hyperparameters—specifically the dropout rate and learning rate—during the training of a ConvLSTM2D-CNN architecture.

The E-HEOA integrates the global exploration strength of the Gaussian-mutated egret swarm optimization algorithm (ESOA) with the local refinement capability of the Cauchy-mutated human evolution optimization algorithm (HEOA). This synergy tackles a predominant challenge in conventional optimizers: maintaining a high convergence rate without compromising the thoroughness of the global search. The incorporation of Gaussian mutation boosts randomness and diversity within the search domain, while Cauchy mutation applies stochastic perturbations to solution vectors, thereby significantly diminishing the probability of the hyperparameter optimization process becoming trapped in local optima.

Furthermore, the model architecture embeds a ConvLSTM2D layer within each hidden tier of the E-HEOA. This design enables convolutional computations to occur at every stage of the forward propagation, significantly fortifying the network’s capacity to process spatiotemporal information and, as a result, refining output image clarity. The experimental analysis leverages a dataset of 170,000 radar echo images and benchmarks performance against eight leading neural networks in the image prediction domain. A novel ConvLSTM2D-CNN model was developed for this purpose. Through two specifically designed gradient experiments, the weight parameters of the ESOA and HEOA were meticulously calibrated to yield a highly effective hybrid optimization strategy. This strategy facilitates the adaptive hyperparameter tuning for the ConvLSTM2D-CNN, culminating in the superior E-HEOA model.

To demonstrate the superiority of the E-HEOA, its performance was compared against ten mainstream evolutionary algorithms, each tasked with the adaptive hyperparameter tuning of the ConvLSTM2D-CNN. The evaluation involved five distinct metrics to assess the prediction outcomes of all ten benchmark models and the proposed E-HEOA. Additionally, the loss function trajectories were analyzed to gauge the convergence speed and fitting performance of each model. The experimental findings confirm that the E-HEOA performs accurate radar echo image extrapolation, offering a valuable tool for engineering applications and practical implementations in radar extrapolation.

Future research directions will focus on two main areas: improving model tuning methods and exploring other radar imaging applications. In terms of enhancing model tuning methods, efforts will center on investigating adaptive tuning approaches to optimize the joint weights within the joint optimization framework. This involves using additional optimization algorithms to fine-tune the weight parameters independently, thereby achieving full automation in the entire joint optimization process. Regarding the exploration of other radar imaging applications, emphasis will be placed on evaluating the stability of the model’s predictive performance for extreme rainfall events and intense convective echoes. To expand the scope of research in these areas, adjustments across four key aspects—data modeling, network architecture, training strategies, and evaluation metrics—are essential. For instance, the dataset could be supplemented with radar data on extreme weather events, and the sampling method could shift from frame-by-frame extraction to overlapping sliding-window sampling. Additionally, the model demonstrates strong extensibility to applications such as traffic prediction, video prediction, and temperature forecasting. Exploring these diverse directions will significantly enhance the model’s generalization capabilities.

## Figures and Tables

**Table 1:** Parameter settings for the neural network.

Parameter Type	Hyperparameter			Input Parameter			Other Parameters	
	Learning Rate	Dropout Rate	Batch size	Epoch	Image Size		Iteration	Population Size
					Length	Width		
Numerical value	0.001	0.2	20	30	64	64	50	100
Unit	\	\	\	\	px	px	\	\

**Table 2:** The evaluation of various models.

Neural Networks	MSE	SSIM
3D-CNN	27.1077	0.6169
E3D-LSTM	51.6478	0.3208
PredRNN++	30.5526	0.6650
ConvGRU	45.961	0.3687
EXT-GAN	65.3061	0.0737
TCN	27.9767	0.7066
CNN-GRU	54.8167	0.3095
CNN-LSTM	58.0365	0.2630
MIM	30.2154	0.6566
TrajGRU	32.4789	0.2431
Earthformer	28.4932	0.1270

**Table 3:** The evaluation results of various models including the new model.

Neural Networks	MSE	SSIM
ConvLSTM2D-CNN	21.5041	0.8006
3D-CNN	27.1077	0.6169
E3D-LSTM	51.6478	0.3208
PredRNN++	30.5526	0.6650
ConvGRU	45.961	0.3687
EXT-GAN	65.3061	0.0737
TCN	27.9767	0.7066
CNN-GRU	54.8167	0.3095
CNN-LSTM	58.0365	0.2630
MIM	30.2154	0.6566
TrajGRU	32.4789	0.2431
Earthformer	28.4932	0.1270

**Table 4:** The associated algorithm evaluation results of each weight parameter with a gradient of 0.1.

Weight	MSE	SSIM	Threshold											
			15 (dbz)			20 (dbz)			30 (dbz)			40 (dbz)		
			POD	CSI	FAR	POD	CSI	FAR	POD	CSI	FAR	POD	CSI	FAR
0.0	19.8641	0.8020	0.9086	0.7363	0.2044	0.9026	0.7289	0.2088	0.8796	0.6993	0.2261	0.8547	0.6763	0.2335
0.1	20.0876	0.8050	0.9093	0.7381	0.2029	0.9024	0.7292	0.2086	0.8847	0.6997	0.2300	0.8624	0.6770	0.2401
0.2	20.1245	0.8073	0.9107	0.7397	0.2023	0.9037	0.7314	0.2073	0.8876	0.7005	0.2314	0.8679	0.6772	0.2445
0.3	20.1316	0.8087	0.9103	0.7394	0.2026	0.9023	0.7303	0.2077	0.8891	0.7014	0.2316	0.8721	0.6772	0.2481
0.4	20.3611	0.8092	0.9096	0.7399	0.2016	0.9015	0.7292	0.2087	0.8909	0.7034	0.2309	0.8759	0.6772	0.2512
0.5	20.1445	0.8092	0.9082	0.7398	0.2008	0.9000	0.7299	0.2068	0.8896	0.7012	0.2329	0.8784	0.6771	0.2534
0.6	20.1836	0.8085	0.9053	0.7382	0.2007	0.8985	0.7299	0.2057	0.8893	0.7011	0.2330	0.8793	0.6766	0.2549
0.7	20.0640	0.8073	0.9023	0.7368	0.2000	0.8967	0.7299	0.2045	0.8881	0.6999	0.2337	0.8792	0.6752	0.2567
0.8	20.2581	0.8054	0.8999	0.7369	0.1982	0.8952	0.7293	0.2040	0.8861	0.6981	0.2346	0.8788	0.6738	0.2584
0.9	20.1561	0.8034	0.8989	0.7380	0.1960	0.8934	0.7279	0.2046	0.8852	0.6974	0.2349	0.8772	0.6714	0.2603
1.0	20.3539	0.8021	0.8949	0.7354	0.1960	0.8898	0.7260	0.2040	0.8810	0.6964	0.2327	0.8758	0.6718	0.2588

**Table 5:** The associated algorithm evaluation results of each weight parameter with a gradient of 0.01.

Weight	MSE	SSIM	Threshold											
			15 (dbz)			20 (dbz)			30 (dbz)			40 (dbz)		
			POD	CSI	FAR	POD	CSI	FAR	POD	CSI	FAR	POD	CSI	FAR
0.40	20.3611	0.8092	0.9096	0.7399	0.2016	0.9015	0.7292	0.2087	0.8909	0.7034	0.2309	0.8759	0.6772	0.2512
0.41	20.3515	0.8092	0.9093	0.7395	0.2018	0.9015	0.7293	0.2086	0.8905	0.7029	0.2312	0.8756	0.6769	0.2514
0.42	20.4270	0.8092	0.9087	0.7394	0.2016	0.9011	0.7292	0.2084	0.8905	0.7030	0.2311	0.8756	0.6770	0.2513
0.43	20.2802	0.8093	0.9087	0.7397	0.2012	0.9004	0.7286	0.2086	0.8905	0.7029	0.2312	0.8768	0.6775	0.2516
0.44	20.3744	0.8093	0.9082	0.7392	0.2015	0.9004	0.7288	0.2083	0.8905	0.7028	0.2313	0.8775	0.6772	0.2525
0.45	20.4303	0.8094	0.9080	0.7391	0.2014	0.9004	0.7294	0.2076	0.8905	0.7028	0.2314	0.8775	0.6769	0.2528
0.46	20.3654	0.8093	0.9082	0.7391	0.2015	0.9002	0.7294	0.2075	0.8900	0.7026	0.2313	0.8780	0.6769	0.2532
0.47	20.1328	0.8093	0.9082	0.7393	0.2013	0.9004	0.7299	0.2071	0.8902	0.7021	0.2322	0.8780	0.6763	0.2539
0.48	19.9989	0.8093	0.9080	0.7395	0.2010	0.8998	0.7296	0.2069	0.8900	0.7019	0.2323	0.8780	0.6762	0.2541
0.49	20.1418	0.8093	0.9080	0.7400	0.2005	0.8998	0.7295	0.2071	0.8894	0.7014	0.2324	0.8784	0.6766	0.2540
0.50	20.1445	0.8092	0.9082	0.7398	0.2008	0.9000	0.7299	0.2068	0.8896	0.7012	0.2329	0.8784	0.6771	0.2534

**Table 6:** The evaluation of predication results for the ConvLSTM2D-CNN model with hyperparameter adaptive optimization based on different algorithms.

Algorithm	MSE	SSIM	Threshold											
			15 (dbz)			20 (dbz)			30 (dbz)			40 (dbz)		
			POD	CSI	FAR	POD	CSI	FAR	POD	CSI	FAR	POD	CSI	FAR
HEOA	21.6842	0.7898	0.9081	0.7012	0.2496	0.9043	0.6925	0.2589	0.8987	0.6684	0.2847	0.8952	0.6421	0.3098
ESOA	20.9176	0.7954	0.8862	0.7186	0.2143	0.8805	0.7089	0.2237	0.8716	0.6792	0.2514	0.8659	0.6531	0.2769
CFOA	38.1829	0.5713	0.9761	0.4995	0.4943	0.9735	0.5320	0.4604	0.9714	0.5699	0.4205	0.9664	0.5680	0.4207
BKA	25.6000	0.7196	0.9607	0.6356	0.3473	0.9624	0.6370	0.3471	0.9624	0.6200	0.3649	0.5940	0.9560	0.3895
APO	19.8641	0.8020	0.9086	0.7363	0.2044	0.9026	0.7289	0.2088	0.8796	0.6993	0.2261	0.8547	0.6763	0.2335
PKO	60.4088	0.3068	0.9870	0.3492	0.6492	0.9874	0.3670	0.6314	0.9854	0.4047	0.5932	0.9839	0.4445	0.5527
AO	40.9203	0.4901	0.9689	0.5571	0.4313	0.9664	0.5960	0.3908	0.9581	0.6146	0.3685	0.9479	0.6037	0.3761
FLO	23.4938	0.7108	0.9015	0.6399	0.3135	0.9046	0.6479	0.3069	0.9072	0.6345	0.3233	0.9070	0.6029	0.3588
PEOA	77.684	0.2569	0.9901	0.2781	0.7205	0.9905	0.2958	0.7028	0.9887	0.3294	0.6687	0.9881	0.3780	0.6202
HO	84.7856	0.1709	0.9904	0.3073	0.6918	0.9901	0.3405	0.6583	0.9906	0.404	0.5944	0.9844	0.4506	0.5464
E-HEOA	19.9989	0.8093	0.9080	0.7395	0.2010	0.8998	0.7296	0.2069	0.8900	0.7019	0.2323	0.8780	0.6762	0.2541

**Table 7:** Evaluation of ablation experiments.

Algorithm	MSE	SSIM	Threshold											
			15 (dbz)			20 (dbz)			30 (dbz)			40 (dbz)		
			POD	CSI	FAR	POD	CSI	FAR	POD	CSI	FAR	POD	CSI	FAR
Unoptimized	21.5041	0.8006	0.8650	0.6802	0.2805	0.8523	0.6621	0.2978	0.8304	0.6315	0.3259	0.8056	0.6024	0.3517
HEOA	21.6842	0.7898	0.9081	0.7012	0.2496	0.9043	0.6925	0.2589	0.8987	0.6684	0.2847	0.8952	0.6421	0.3098
GM-HEOA	21.3223	0.7910	0.9090	0.7101	0.2422	0.9056	0.7038	0.2513	0.8994	0.6718	0.2783	0.8966	0.6456	0.3038
CM-HEOA	21.1503	0.7965	0.9174	0.7188	0.2314	0.9155	0.7107	0.2400	0.9108	0.6851	0.2660	0.9086	0.6609	0.2922
ESOA	20.9176	0.7954	0.8862	0.7186	0.2143	0.8805	0.7089	0.2237	0.8716	0.6792	0.2514	0.8659	0.6531	0.2769
CM-ESOA	21.0824	0.7982	0.8901	0.7230	0.2073	0.8832	0.7135	0.2176	0.8756	0.6851	0.2483	0.8693	0.6559	0.2668
GM-ESOA	20.3539	0.8021	0.8949	0.7354	0.1960	0.8898	0.7260	0.2040	0.8810	0.6964	0.2327	0.8758	0.6718	0.2588
E-HEOA	19.9989	0.8093	0.9080	0.7395	0.2010	0.8998	0.7296	0.2069	0.8900	0.7019	0.2323	0.8780	0.6762	0.2541

**Table 8:** Evaluation of short-term forecast performance.

Lead Time (min)	MSE	SSIM	Threshold											
			15 (dbz)			20 (dbz)			30 (dbz)			40 (dbz)		
			POD	CSI	FAR	POD	CSI	FAR	POD	CSI	FAR	POD	CSI	FAR
30	19.829	0.8148	0.9126	0.7448	0.1967	0.9039	0.7341	0.2028	0.8942	0.7078	0.2289	0.8821	0.6816	0.2493
60	19.9989	0.8093	0.9080	0.7395	0.2010	0.8998	0.7296	0.2069	0.8900	0.7019	0.2323	0.8780	0.6762	0.2541
90	22.47	0.7816	0.8764	0.6941	0.2786	0.8619	0.6687	0.3042	0.8338	0.6325	0.3417	0.8012	0.5968	0.3789
120	25.08	0.7524	0.8421	0.6528	0.3154	0.8217	0.6234	0.3439	0.7896	0.5861	0.3828	0.7543	0.5497	0.4196

**Table 9:** Evaluation of time complexity.

Algorithm	Computational Complexity
HEOA	$\Theta \left(T_{1}\times M\right)$
ESOA	$\Theta \left(T_{2}\times M\right)$
GM-HEOA	$\Theta \left(T_{3}\times M\right)$
CM-ESOA	$\Theta \left(T_{4}\times M\right)$
E-HEOA	$\Theta \left(T_{5}\times M\right)$

**Table 10:** Evaluation of E-HEOA and ConvLSTM2D-CNN under different noise thresholds.

Method	SSIM				
	Noise-free	15 (dbz)	20 (dbz)	30 (dbz)	40 (dbz)
ConvLSTM2D-CNN	0.8006	0.794	0.7871	0.7788	0.7712
E-HEOA	0.8093	0.8049	0.8006	0.7949	0.7886

**Table 11:** Significance testing of SSIM improvement of E-HEOA compared to ConvLSTM2D-CNN.

Method	Mean Value	Standard Deviation	Sample Size	t-Value	*p*-Value
ConvLSTM2D-CNN	0.8003	0.0035	10	7.19	*p* < 0.001
E-HEOA	0.8105	0.0028			

**Table 12:** Sensitivity analysis of key parameters.

Population	Iteration	MSE	SSIM	Threshold											
				15 (dbz)			20 (dbz)			30 (dbz)			40 (dbz)		
				POD	CSI	FAR	POD	CSI	FAR	POD	CSI	FAR	POD	CSI	FAR
25	50	26.8421	0.7214	0.8612	0.6418	0.2841	0.8486	0.6289	0.2917	0.8215	0.6021	0.3184	0.7924	0.5712	0.3469
50	50	24.9136	0.7428	0.8745	0.6629	0.2663	0.8617	0.6498	0.2738	0.8351	0.6216	0.3012	0.8068	0.5894	0.3297
75	50	23.2048	0.7639	0.8873	0.6845	0.2479	0.8752	0.6713	0.2551	0.8487	0.6428	0.2836	0.8214	0.6107	0.3122
100	50	22.1179	0.7816	0.8958	0.7034	0.2312	0.8841	0.6896	0.2394	0.862	0.6619	0.2679	0.8352	0.6298	0.2965
125	50	22.0045	0.7841	0.8969	0.7051	0.2298	0.8853	0.6912	0.238	0.8631	0.6635	0.2664	0.8364	0.6312	0.295
25	100	23.9147	0.7549	0.8816	0.6732	0.2584	0.8698	0.6601	0.2659	0.8427	0.6325	0.2931	0.8149	0.6008	0.3214
50	100	22.0083	0.7746	0.8937	0.6945	0.2416	0.8824	0.6819	0.2487	0.8559	0.6541	0.2762	0.8286	0.6224	0.3046
75	100	20.6842	0.7958	0.9041	0.7158	0.2239	0.8936	0.7034	0.2311	0.8678	0.6759	0.2586	0.8412	0.6441	0.2873
100	100	20.1126	0.8034	0.9073	0.7321	0.2108	0.8971	0.7194	0.2189	0.8769	0.6928	0.2457	0.853	0.6609	0.2744
125	100	20.0879	0.8041	0.9079	0.733	0.2101	0.8978	0.7201	0.2182	0.8775	0.6935	0.245	0.8536	0.6616	0.2738
25	150	22.3041	0.7723	0.8921	0.6994	0.2368	0.8803	0.6862	0.2439	0.8536	0.6587	0.2708	0.8259	0.6268	0.2989
50	150	20.9876	0.7918	0.9019	0.7216	0.2197	0.8912	0.7089	0.2268	0.8654	0.6812	0.2539	0.8396	0.6495	0.2824
75	150	20.2148	0.8037	0.9059	0.7341	0.2109	0.8956	0.7215	0.2181	0.8731	0.6948	0.2446	0.8482	0.6632	0.2731
100	150	19.9989	0.8093	0.908	0.7395	0.2010	0.8998	0.7296	0.2069	0.8900	0.7019	0.2323	0.8780	0.6762	0.2541
125	150	20.0114	0.8087	0.9075	0.7399	0.2005	0.9006	0.7299	0.2060	0.8894	0.7011	0.2330	0.8774	0.6755	0.2548
25	200	22.5118	0.7709	0.8913	0.6986	0.2375	0.8794	0.6854	0.2447	0.8527	0.6579	0.2716	0.8249	0.626	0.2996
50	200	21.1024	0.7906	0.9011	0.7208	0.2204	0.8904	0.7081	0.2276	0.8646	0.6804	0.2546	0.8387	0.6487	0.2831
75	200	20.3189	0.8026	0.9052	0.7334	0.2116	0.8949	0.7208	0.2188	0.8724	0.6941	0.2453	0.8475	0.6625	0.2738
100	200	20.0012	0.8089	0.9078	0.739	0.2018	0.8994	0.7291	0.2076	0.8896	0.7013	0.2332	0.8776	0.6757	0.255
125	200	19.9985	0.8087	0.9082	0.7394	0.2014	0.8993	0.7285	0.2082	0.8890	0.7017	0.2328	0.8782	0.6761	0.2536
25	250	22.6084	0.7698	0.8907	0.6979	0.2381	0.8788	0.6847	0.2453	0.8521	0.6573	0.2722	0.8243	0.6254	0.3002
50	250	21.1847	0.7899	0.9006	0.7202	0.221	0.8899	0.7075	0.2282	0.8641	0.6798	0.2552	0.8381	0.6481	0.2837
75	250	20.3926	0.8019	0.9048	0.7328	0.2122	0.8944	0.7202	0.2194	0.8719	0.6936	0.2459	0.847	0.662	0.2744
100	250	19.9876	0.8081	0.9083	0.7385	0.2003	0.8989	0.7286	0.2051	0.8891	0.7008	0.2337	0.8791	0.6764	0.2535
125	250	19.9880	0.8086	0.9078	0.7390	0.2009	0.9010	0.7291	0.2057	0.8886	0.7003	0.2343	0.8786	0.6767	0.2535

## Data Availability

The raw data supporting the conclusions of this article will be made available by the corresponding author on request.
